# Effect of Different Basal Culture Media and Sera Type Combinations on Primary Broiler Chicken Muscle Satellite Cell Heterogeneity during Proliferation and Differentiation

**DOI:** 10.3390/ani12111425

**Published:** 2022-05-31

**Authors:** Joshua J. Flees, Charles W. Starkey, Jessica D. Starkey

**Affiliations:** Department of Poultry Science, Auburn University, Auburn, AL 36849, USA; jjf0021@auburn.edu (J.J.F.); cstarkey@auburn.edu (C.W.S.)

**Keywords:** primary muscle satellite cell, broiler chicken, cell culture media, serum, doubling time, myoblast fusion, myogenic regulatory factor expression

## Abstract

**Simple Summary:**

Little consistency in the literature exists for optimal culture conditions for proliferating and differentiating primary broiler chicken muscle satellite cells regarding basal culture media, proliferation sera, and differentiation sera. This experiment assessed primary satellite cell proliferation and differentiation when cultured in different combinations of basal media and sera. Cells were cultured in different basal media: low glucose Dulbecco’s Modified Eagle’s medium, McCoy’s 5A, and high glucose Dulbecco’s Modified Eagle’s medium. Each media was supplemented with 15% chicken serum, or a combination of 5% horse serum + 10% chicken serum during proliferation while 3% horse serum or 3% chicken serum were supplemented during differentiation. Cultures were immunofluorescence stained for myogenic regulatory factors at different time points during proliferation and differentiation. During proliferation and differentiation, cells cultured in Dulbecco’s Modified Eagle’s medium tended to have higher proportions of myogenic cells expressing myogenic regulatory factors and promoted satellite cell fusion into myotubes compared with McCoy’s 5A. Low glucose media, glucose concentration similar to circulating glucose concentrations in broilers, combined with sera published in the literature may be the optimal culture media to promote satellite cell proliferation and differentiation.

**Abstract:**

The objective of this experiment was to access primary satellite cell (SC) proliferation and differentiation when cultured in different combinations of basal media and sera due to little consistency being published on the optimal culture media for primary broiler chicken satellite cells. Cells were cultured in one of three different basal media: McCoy’s 5A, high glucose Dulbecco’s Modified Eagle’s medium (DMEM), and low glucose DMEM. Media were supplemented with 15% chicken serum (CS) or a combination of 5% horse serum (HS) + 10% CS during proliferation while 3% HS or 3% CS were added to the media during differentiation. Cultures were immunofluorescence stained for myogenic regulatory factors (MRF) at 48, 72, and 96 h post-plating for proliferation (Pax7, MyoD, and Myf-5) and 96 h post-proliferation during differentiation (Pax7 and MyoD), including MF20 to assess fusion. Cells cultured in Dulbecco’s Modified Eagle’s medium tended to have higher proportions of myogenic cells expressing MRF during proliferation and promoted fusion into myotubes compared with McCoy’s 5A during differentiation. Culturing primary SC in low glucose media, glucose concentrations similar to circulating glucose concentrations in broilers, HSCS during proliferation and CS during differentiation, appears to be optimal for promoting broiler chicken satellite cell proliferation and differentiation.

## 1. Introduction

Muscle satellite cells (SC), within skeletal muscle tissue, are muscle specific stem cells residing between the sarcolemma and basal lamina of muscle fibers [1] and are necessary for skeletal muscle development and growth. At the start of muscle development, myoblasts fuse to form multinucleated myotubes to later differentiate into muscle fibers; however, the number of muscle fibers is fixed at hatch in birds [2] and hypertrophy of post-mitotic muscle fibers occurs during post-hatch growth of the animal. Activated SC will proliferate and fuse with growing muscle fibers, contributing their DNA for myofibrillar protein synthesis; thus, increasing muscle fiber cross sectional area [3,4]. Due to their unique function, SC isolated from *Pectoralis major* muscle are useful for in vitro experiments for studying their functions regarding muscle growth by assessing their ability to proliferate and differentiate in culture.

In most studies, C2C12 (mouse) or L6 (rat) cell lines are utilized in experiments compared with primary SC due to primary cultures containing a mixed population of myogenic and non-myogenic cells if pre-plating or centrifugation is not applied to remove the non-myogenic populations during isolation [5]. However, primary SC cultures represent a model that closer represents in vivo conditions for production animals. Early and modern studies utilizing SC for experiments optimized isolation and culture conditions for SC isolated from bovine [6,7,8], ovine [9], porcine [10], avian [11], and murine sources [12]. McFarland and colleagues optimized culture conditions for turkey SC evaluating 36 different media-sera combinations during SC proliferation and differentiation, which revealed that different media and sera can impact SC proliferation and differentiation [11]. However, no publications regarding optimal culture conditions for broiler chickens have been published to our knowledge and there are inconsistencies in the literature between different studies for proliferation and differentiation media [13,14,15].

Therefore, this study was undertaken to evaluate the effect of different basal culture media and sera type combinations of primary broiler chicken satellite cells during proliferation and differentiation.

## 2. Materials and Methods

### 2.1. Muscle Satellite Cell Isolation

Skeletal muscle SC were isolated from *Pectoralis major* tissue of eight, 22-day-old, straight run, Yield Plus × Ross 708 broilers utilizing a protocol adapted from previously published studies [6,11]. After euthanasia by carbon dioxide asphyxiation and cervical dislocation, broilers were sprayed with a 70% ethanol solution and placed on a dissection tray. With the muscle oriented dorsally, the skin was retracted, and *Pectoralis major* tissue was removed using sterile tools. Muscle tissue was placed on a sterile surgical tray containing 5% 20X phosphate-buffered saline in ultra-pure water solution (PBS; Cat. No. 10010-072, Gibco Life Technologies, Grand Island, NY, USA) supplemented with 1% antibiotic/antimycotic (Cat. No. 15240, Gibco Life Technologies, Grand Island, NY, USA) and 0.1% gentamicin (Cat. No. 15750, Gibco Life Technologies, Grand Island, NY, USA). In a laminar flow hood, muscle tissue was ground using a sterile meat grinder with a medium die onto a sterile dissection tray. Six 50 mL conical-bottom centrifuge tubes (Cat. No. 89039-658, VWR International, Radnor, PA, USA) were filled with ground muscle tissue and sterile filtered 1 mg per mL pronase protease solution (Cat. No. 53702, Calbiochem, San Diego, CA, USA) at 1.5 mL of pronase solution to 1 g of tissue. Muscle tissue was incubated in the pronase solution to facilitate tissue digestion for 30 min in a non-shaking 40 °C water bath while slowly inverting the conicals every 10 min. After tissue digestion, conicals were spun at 1500× *g* for 4 min in a centrifuge to pellet the tissue and to discard the pronase solution. After discarding the protease solution, PBS supplemented with 1% antibiotic/antimycotic + 0.1% gentamicin (2 mL of PBS solution per 1 g of tissue) was added to each conical at 2 mL of PBS per 1 g of tissue, and tissue was resuspended into solution to rinse the tissue of any remaining pronase solution. The conicals were centrifuged again at 1500× *g* for 10 min and the supernatant was discarded. After, PBS supplemented with 1% antibiotic/antimycotic + 0.1% gentamicin was added, tissue was resuspended into solution, and conicals were centrifuged for 500× *g* for 10 min. The resulting supernatant containing cells was transferred into new conicals and centrifuged for 1500× *g* for 10 min to pellet the cells. The supernatant was aspirated off and cells were resuspended in room temperature, high glucose Dulbecco’s Modified Eagle medium (HGDMEM; Cat. No. 11965-118, Life Technologies, Grand Island, NY, USA) supplemented with 10% horse serum (HS; Cat. No. SH30074.03, Thermo Fischer Scientific, Fairlawn, NJ, USA) and 3% antibiotic/antimycotic + 0.3% gentamicin. The process was repeated 2 additional times using the same muscle tissue in the 6 conicals until 3 replicate conicals of cells were generated. All 3 conicals of cells were combined into 1 new conical and slowly filtered using a 100 µm and 40 µm Steriflip Vacuum-driven Filtration Systems conical filter (Cat. No. SCNY00100 and SCNY00040, respectively, MilliporeSigma, Darmstadt, Germany). The cell suspensions for each of the 6 conicals was evenly distributed between 2 new 50 mL conicals containing HGDMEM supplemented with 10% HS and 3% antibiotic/antimycotic + 0.3% gentamicin before being plated evenly among four 182.5 cm^2^ tissue culture flasks. Additional media was added to each flask and flasks were incubated in a 40 °C humidified incubator at 5% carbon dioxide and 18% oxygen for 2 h to plate any non-SC populations. All unattached cells in suspension were removed from the flask and placed into 4 new 50 mL conicals. The cells were centrifuged at 1500× *g* for 10 min, the supernatant was aspirated off, and the conicals were placed on ice. Cells were resuspended in ice cold HGDMEM supplemented with 10% HS and gently mixed after the addition of ice cold HGDMEM supplemented with 10% HS + 20% dimethyl sulfoxide (Cat. No. D2650, MilliporeSigma, Darmstadt, Germany) for a final concentration of 10% dimethyl sulfoxide in the cell suspension. The cell suspension was evenly aliquoted into cryovials to a final concentration of 6 g of tissue per cryovial and placed at −80 °C overnight and then stored in a liquid nitrogen tank until the beginning of the experiment.

### 2.2. Satellite Cell Culture Conditions

Cryovials of frozen cells (n = 24) were removed from liquid nitrogen prior to plating and allowed to partially thaw in a 40 °C water bath. Vials were divided between 3 replicate conicals (n = 8 vials per replicate) of warm, serum-free, low glucose Dulbecco’s Modified Eagle’s medium (LGDMEM; Cat. No. 10569-010, Life Technologies, Grand Island, NY, USA). A small aliquot of the cell suspension from each replicate was used to determine the number of cells by using a hemacytometer. Cells were plated at 1.66 × 10^6^ cells per well on 15 separate 24-well tissue culture plates (Cat. No.m10062-896: VWR International, Radnor, PA, USA) (n = 5 plates per replicate). All wells were coated with 0.1% gelatin (Cat. No. ES-006-B, MilliporeSigma, Darmstadt, Germany) per the manufacturer’s instructions prior to cell plating. The 24 wells per plate were divided into 6 blocks of 4 wells to represent 1 of 6 different basal culture media and supplemental sera combinations for culture. The 3 basal culture media utilized were: (1) LGDMEM, (2) McCoy’s 5A (MCCOY; Cat. No. 16600-082, Life Technologies, Grand Island, NY, USA), and (3) HGDMEM (Cat. No. 10567-014, Life Technologies, Grand Island, NY, USA). The composition of the 3 basal culture medias is shown in Supplemental Table S1. The sera supplemented into media for satellite cell proliferation were: (1) 15% chicken serum (CS; Cat. No. C5405, Millipore Sigma, Darmstadt, Germany) or (2) a combination of 5% HS and 10% CS (HSCS). A complete proliferation media consisted of 1 basal culture media, 1 serum, and 1% antibiotic/antimycotic (Cat. No. 15240-062, Life Technologies, Grand Island, NY, USA) + 0.1% gentamicin (Cat. No. 15710-064, Life Technologies, Grand Island, NY, USA) and all wells were fed the same media for the entirety of proliferation. All parallel plates were incubated at 40 °C with 5% carbon dioxide and 18% oxygen in a humidified New Brunswick Galaxy 170 R tri-gas incubator (Eppendorf, Hamburg, Germany). After 48 h post-plating, plates were rinsed twice with serum free media, respective to their basal culture media treatment, and fed fresh proliferation media. At 48, 72, and 96 h post-plating, one plate per replicate was rinsed twice with serum free media and incubated in cold methanol for 10 min for cell fixation. The 3 plates at each time point were immediately subjected to indirect immunofluorescence (IF) staining described below.

After 96 h post-plating the 2 remaining plates per replicate were rinsed twice with serum-free media, and fed with differentiation media for 96 h. Cells were differentiated in the same basal culture media during proliferation but supplemented with 2 different sera for differentiation: (1) 3% CS or (2) 3% HS. At 48 h post-proliferation, plates were rinsed with serum-free media, respective to their treatments, and fed fresh differentiation media. After 96 h post-proliferation, plates were rinsed twice with serum free media, incubated in cold methanol for 10 min for cell fixation to the plates, and immediately IF stained for assessment of myotube and unfused SC heterogeneity at 96 h post-proliferation.

### 2.3. Indirect Immunofluorescence Staining

Cultures at 48, 72, and 96 h post-plating from the proliferation assay and 96 h post-proliferation from the differentiation assay were indirect immunofluorescence stained for myogenic regulatory factors (MRF) using a previously published protocol [16] with minor modifications. Briefly, methanol fixed cells were rinsed 2 times with PBS and blocked with a blocking solution containing PBS + 5% HS (Thermo Fisher Scientific, Fairlawn, NJ, USA) + 0.2% Triton-X100 (Cat. No. 85112; Thermo Fisher Scientific, Fairlawn, NJ, USA) for 30 min at room temperature. Primary and secondary antibodies (described below) were diluted in a blocking solution prior to being applied to plates. All plates were rinsed and washed with PBS three times for 5 min after primary (90 min) and secondary (30 min) incubations at room temperature. All cell nuclei were counterstained with 4′-6-diamidino-2-phenylindole (DAPI; Thermo Fisher Scientific, Waltham, MA, USA) diluted in PBS to a final concentration of 1 µg per mL for 1 min with a final PBS rinse before imaging.

The primary antibodies used on proliferation plates were as follows: mouse IgG1 anti-Pax7 (Pax7; Cat. No. PAX7, Developmental Studies Hybridoma Bank, Iowa City, IA, USA) hybridoma cell supernatant diluted 1:10, mouse monoclonal IgG2b anti-myogenic differentiation factor 1 (MyoD; Cat. No. SC-377460, Santa Cruz Biotechnology, Dallas, TX, USA) diluted 1:2000, and rabbit polyclonal IgG anti-myogenic factor 5 (Myf-5; Cat. No. SC-302, Santa Cruz Biotechnology, Dallas, TX, USA) diluted 1:350. The primary antibodies used for differentiation included: mouse IgG2b anti-MF20 (MF20; Cat. No. MF 20, Developmental Studies Hybridoma Bank, Iowa City, IA, USA) hybridoma cell supernatant diluted 1:10, PAX7, and MYOD. Primary antibodies were detected using the following secondary antibodies diluted 1:1000 in blocking solution: Alexa-Fluor 488 conjugated goat anti-mouse IgG1 (Cat. No. A-21121, Invitrogen, Eugene, OR, USA) Alexa-Fluor 546 conjugated goat anti-mouse IgG2b (Cat. No. A-21143, Invitrogen, Eugene, OR, USA), and Alexa-Fluor 633 goat anti-rabbit IgG (Cat. No. A-21070, Invitrogen, Eugene, OR, USA).

### 2.4. Primary Satellite Cell Taxonomy

Fixed culture plates were imaged at 200-fold magnification using an inverted Eclipse Ti-U microscope with an incorporated fluorescence illuminator Epi-FI filter turret and a 365 nm light engine (Nikon Instruments Inc., Melville, NY, USA). Digital captures were taken with an incorporated Evolve 512 EMMCF digital monochrome camera (Teledyne Photometrics, Tucson, AZ, USA). Two representative images per well (n = 8 per treatment) were captured and analyzed using the NIS-Elements: Advanced Research Software (Nikon Instrument Inc., Melville, NY, USA).

To determine heterogeneity of myogenic SC populations during proliferation at 48, 72, and 96 h post-plating, enumeration of total nuclei and all cell populations with MyoD+, Myf-5+, Pax7+, MyoD+: Myf-5+, Myf-5+:Pax7+, MyoD+:Pax7+, and MyoD+:Myf-5+:Pax7+ was performed with the NIS-Elements software Taxonomy tool. All cell populations enumerated were also DAPI+ in addition to their immunofluorescence profile. The total number of DAPI+ nuclei per image were determined in each image as a measure of nuclear density and to determine total DAPI+ cells. All MyoD+, Myf-5+, and Pax7+ cells with or without other MRF markers were considered myogenic SC. The total number of DAPI+:MRF+ nuclei per image were determined in each image as a measure of total MRF+ cells while cells only expressing DAPI+ nuclei (MFR--) were considered total MRF- cells. Proportions of total MRF+ and total MRF- to total DAPI+ cells were determined at 48, 72, and 96 h post-plating during proliferation. The population doubling time (PDT) in d of total MRF+ cells was calculated using the following equation:

$$PDT=\left(t/log_{2}\left(N_{t}/C_{i}\right)\right)\times 24$$

where t = time in d, $N_{t}$ = final cell number, $C_{i}$ = initial cell number [17], and a factor of 24 was used to convert d to h.

To determine heterogeneity of myogenic SC populations during differentiation at 96 h post-proliferation, MF20 was used as a nascent myotube marker to enumerate DAPI+ cells within fused myotubes. Enumeration of total nuclei and all cell populations with MyoD+, Pax7+, and MyoD+:Pax7+ nuclei within myotubes (myonuclei) and outside myotubes (unfused cells) was performed with the NIS-Elements software Taxonomy tool. All cell populations and myonuclei enumerated were also DAPI+ in addition to their immunofluorescence profile. The total number of DAPI+ nuclei per image were determined in each image as a measure of nuclear density and to determine total DAPI+ cells within myotubes and outside fused myotubes. All DAPI+, MyoD+, and Pax7+ nuclei within myotubes were considered myonuclei and the total number of DAPI+:MRF+ myonuclei were measured in each image as a measure of total myonuclei. All DAPI+:MRF+ cells outside myotubes measured in each image as a measure of total unfused, myogenic cells while DAPI+:MRF- cells outside of myotubes were measured in each image as a measure of total MRF- cells. The total unfused, myogenic cells and total MRF- cells were measured in each image as a measure of total non-myonuclei. The total myonuclei, total non-myonuclei, and total MRF- cells were measured in each image as a measure of total nuclei. Percent fusion of myotubes was calculated as a proportion of total myonuclei to total nuclei populations. All population means were expressed as number per mm^2^ basis and population proportions were the ratio of the specific population to total DAPI+ cells.

### 2.5. Statistical Analysis

Proliferation heterogeneity and PDT data were analyzed as a 2-way analysis of variance using the generalized linear mixed model GLIMMIX procedure in SAS version 9.4 (PC version 9.4, SAS Inst. Inc., Cary, NC, USA) where basal culture media, proliferation sera, and basal culture media × proliferation sera were the main effects. Differentiation heterogeneity and fusion data were analyzed as a 3-way analysis of variance using the generalized linear mixed model GLIMMIX procedure where basal culture media, proliferation sera, differentiation sera, basal culture media × proliferation sera, basal culture media × differentiation sera, proliferation sera × differentiation sera, and basal culture media × proliferation sera × differentiation sera were the main effects. Cell culture well (n = 144 for proliferation at 48, 72, and 96 h post-plating each, and n = 288 for differentiation) served as the experimental unit. All possible pairwise least square mean comparisons were performed using the PDIFF option of SAS at *p* < 0.05 and the Satterthwaite adjustment was used to correct the degrees of freedom. Proportional data were analyzed using the events/experiments syntax with a binomial distribution and both continuous and proportional data were analyzed using an R-side covariance structure. Significant differences were declared when *p* ≤ 0.05 and tendencies when 0.0501 ≤ *p* ≤ 0.10.

## 3. Results

### 3.1. Proliferation Heterogeneity and Population Doubling Time

Primary SC heterogeneity of all MRF+ and MRF- cell populations were assessed at 48, 72, and 96 h post-plating when cells were cultured in a combination of one of three basal culture media and one of two proliferation sera. At 48 h post-plating, there was no interaction among basal culture media × proliferation serum on single, double, or triple MRF+ cells (*p* ≤ 0.05; Table 1) except MCCOY media supplemented with HSCS tended to produce more Myf-5+MyoD+:Pax7+ SC compared with all other treatments (*p* = 0.0911; Table 1).

**Figure 1:**
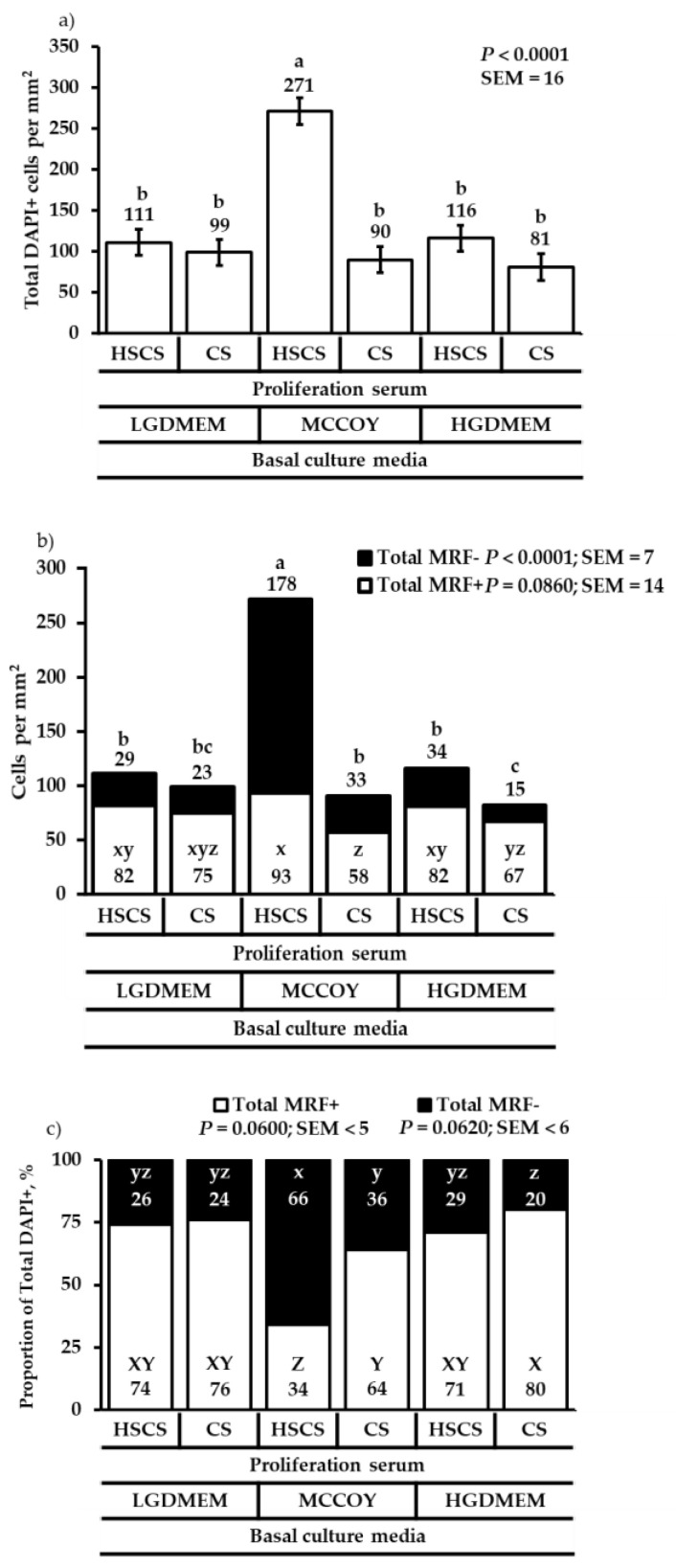
The effect of basal culture media and proliferation sera on total DAPI+ cells (**a**), total MRF+ and total MRF- cells (**b**), and proportions of total MRF+ or total MRF- cells to total DAPI+ cells (**c**) at 48 h post-plating. Cells were cultured in either low glucose Dulbecco’s Modified Eagles medium (LGDMEM), McCoy’s 5A (MCCOY), or high glucose Dulbecco’s Modified Eagles medium (HGDMEM) supplemented with either 5% horse serum + 10% chicken serum (HSCS) or 15% chicken serum (CS). ^a–c^ Means with different superscripts describe a difference *p* < 0.05 and were generated from the pairwise mean comparison analysis. ^x–z^ Means with different superscripts describe a tendency 0.0501 ≤ *p* ≤ 0.10 and were generated from the pairwise mean comparison analysis.

The most cells (total DAPI+ cells) were produced when cells were cultured in MCCOY media supplemented with HSCS compared with other media and sera combinations (*p* < 0.0001; Figure 1a). However, the same media and sera combination produced the more total MRF- cells (*p* < 0.0001; Figure 1b) and tended to produce more total MRF+ cells (*p* = 0.0860; Figure 1b). Even though MCCOY+HSCS media produced the most cells at 48 h post-plating, total MRF+ cells tend to make up greater that 70% of the total DAPI+ cells when cells were cultured in either LGDMEM or HGDMEM regardless of proliferation sera supplemented into the media (*p* ≤ 0.0618; Figure 1c). When cells were cultured in MCCOY+HSCS media, a higher proportion of total MRF- cells to the total DAPI+ cells (*p* = 0.0618; Figure 1c) tended to be produced.

By 72 h post-plating, cells cultured in MCCOY+HSCS media produced the most Pax7+ (*p* = 0.0040; Table 2) and Myf-5+:MyoD+:Pax7+ (*p* < 0.0001; Table 2) compared with other media and sera combinations. Interestingly, the most Myf-5+:MyoD+ cells were produced when cells were cultured in LGDMEM or HGDMEM when supplemented with HSCS compared with MCCOY+HSCS that better supported two other cell populations (*p* = 0.0150; Table 2).

**Figure 2:**
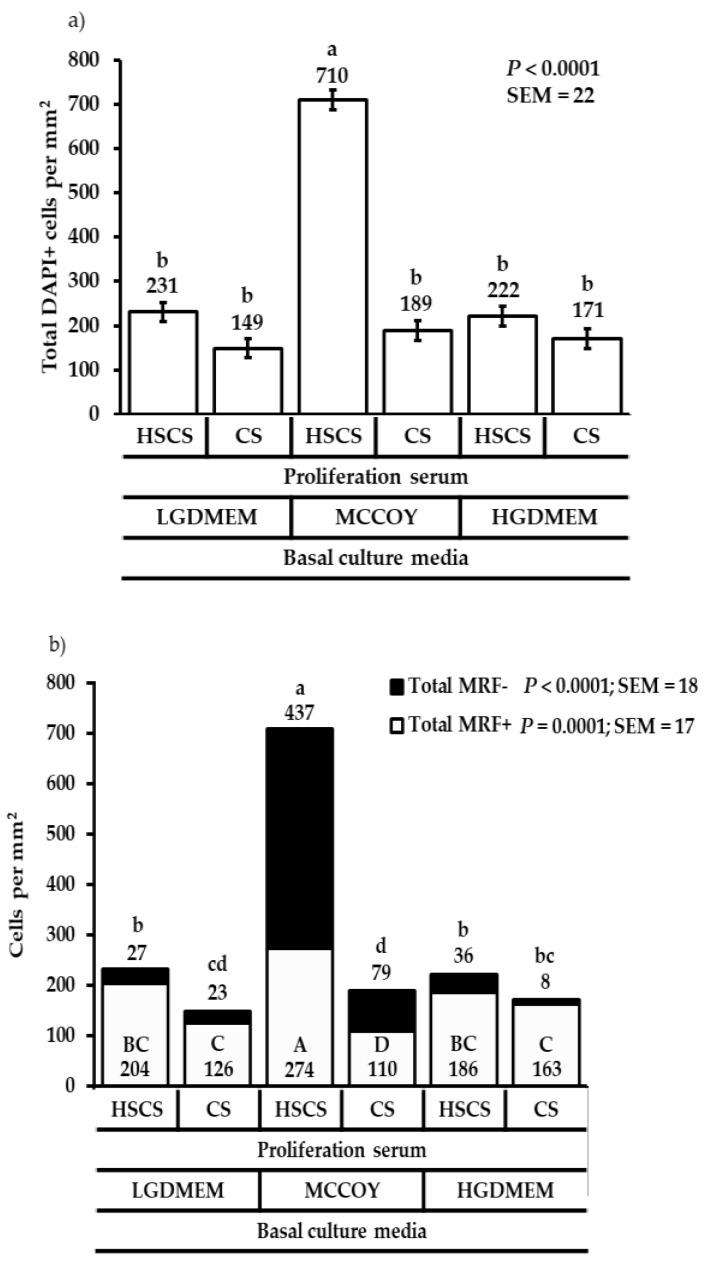

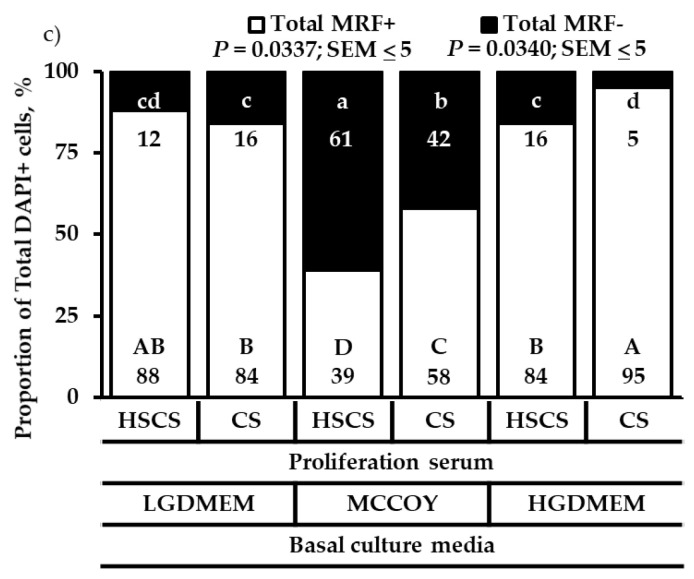
The effect of basal culture media and proliferation sera on total DAPI+ cells (**a**), total MRF+ and total MRF- cells (**b**), and proportions of total MRF+ or total MRF- cells to total DAPI+ cells (**c**) at 72 h post-plating. Cells were cultured in either low glucose Dulbecco’s Modified Eagles medium (LGDMEM), McCoy’s 5A (MCCOY), or high glucose Dulbecco’s Modified Eagles medium (HGDMEM) supplemented with either 5% horse serum + 10% chicken serum (HSCS) or 15% chicken serum (CS). ^a–d; A–D^ Means with different superscripts describe a difference *p* ≤ 0.05 and were generated from the pairwise mean comparison analysis.

Following similar results at 48 h post-plating, the most cells (total DAPI+ cells) were produced when cells were cultured in MCCOY+HSCS compared with the other media and sera combinations (*p* < 0.0001; Figure 2a). The same media and serum combination also produced the most total MRF+ (*p* = 0.0001; Figure 2b), and total MRF- (*p* < 0.0001; Figure 2b) cell populations. It is not surprising that more than 40% of the total cells cultured in MCCOY+HSCS were MRF- with MCCOY+CS having the second highest percentage of MRF- cells (*p* = 0.0340; Figure 2c). Nevertheless, both DMEM media supported MRF+ cell proliferation due to more than 84% of the total cells being MRF+ (*p* = 0.0337; Figure 2c) even though MCCOY+HSCS produced more cells.

At the last timepoint in proliferation media, 96 h post-plating, cells cultured in HGDMEM+HSCS media produced more Myf-5+:MyoD+ cells are (*p* < 0.0001; Table 3) compared with other media and sera combinations.

**Figure 3:**
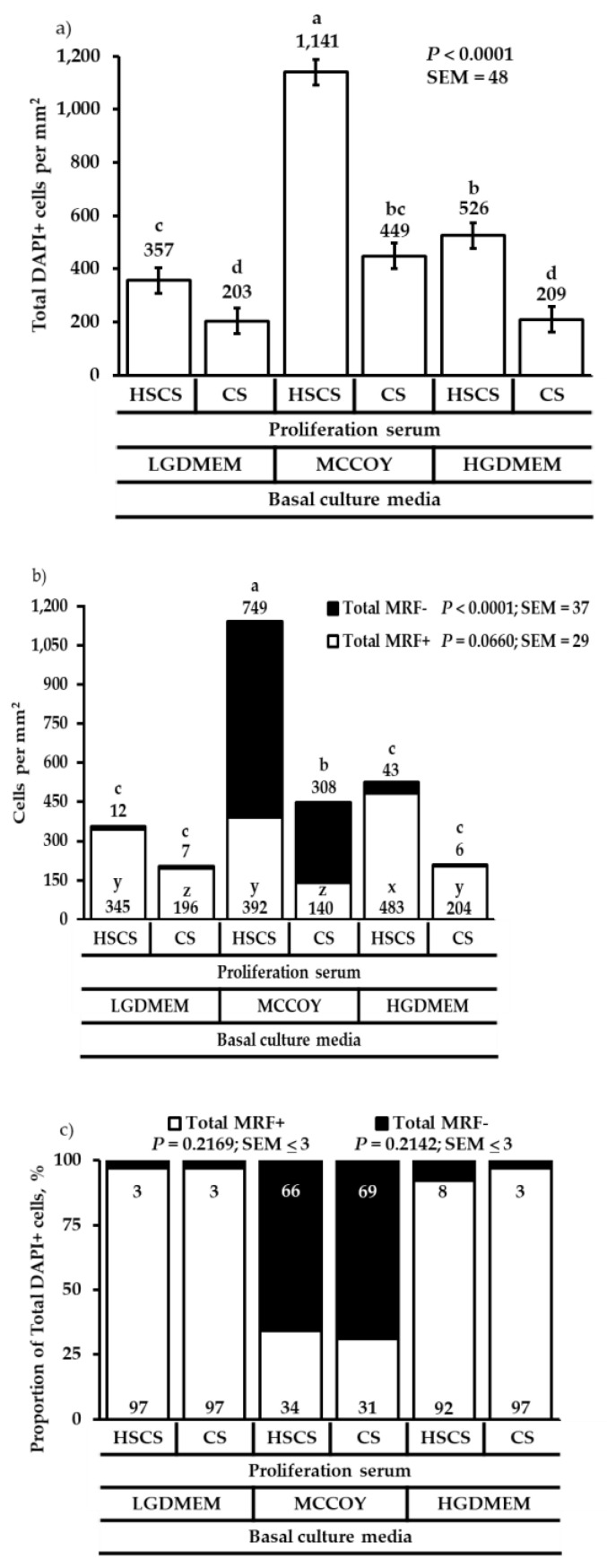

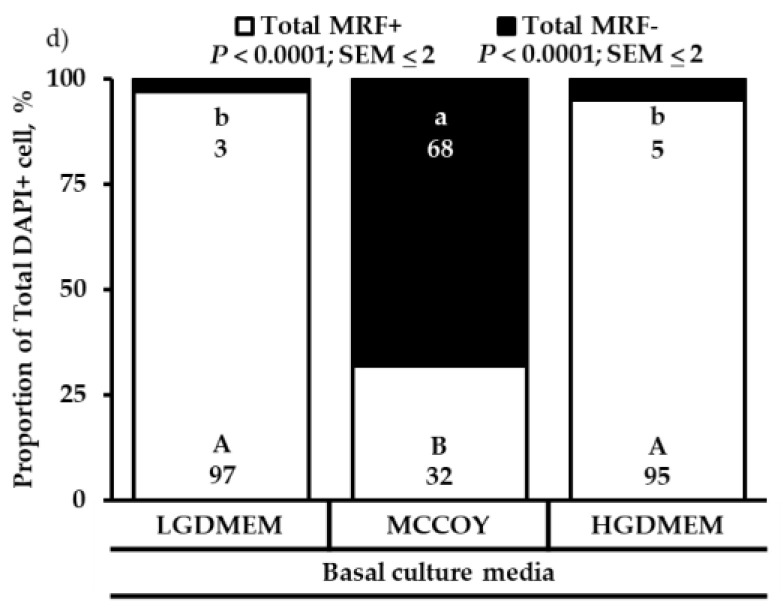
The effect of basal culture media and proliferation sera on total DAPI+ cells (**a**), total MRF+ and total MRF- cells (**b**), proportions of total MRF+ or total MRF- cells to total DAPI+ cells (**c**), and the main effect of basal culture media on total MRF+ or total MRF- cells to total DAPI+ cells at 96 h post-plating (**d**). Cells were cultured in either low glucose Dulbecco’s Modified Eagles medium (LGDMEM), McCoy’s 5A (MCCOY), or high glucose Dulbecco’s Modified Eagles medium (HGDMEM) supplemented with either 5% horse serum + 10% chicken serum (HSCS) or 15% chicken serum (CS). ^a–d^ Means with different superscripts describe a difference *p* < 0.05 and were generated from the pairwise mean comparison analysis. ^x–z^ Means with different superscripts describe a tendency 0.10 < *p* < 0.05 and were generated from the pairwise mean comparison analysis.

Primary SC cultured in MCCOY+HSCS media produced the most total cells and total MRF- cell populations compared with the other media combinations (*p* < 0.0001; Figure 3a,b), which was consistent with results from 48 and 72 h post-plating. Nevertheless, SC cultured in HGDMEM+HSCS media tended to produce more total MRF+ cells with LGDMEM and MCCOY media supplemented with HSCS (*p* = 0.0660; Figure 3b and Supplemental Figure S1). While the ANOVA did not detect a difference in the proportional data for the interaction of basal culture media × sera (*p* > 0.2142; Figure 3c), the main effect of basal culture media revealed that cells cultured in either DMEM media promoted a higher proportion of total MRF+ cells to total DAPI+ cells (*p* < 0.0001; Figure 3d) compared with culturing cells in MCCOY media, which promoted a higher proportion of total MRF- cells to total DAPI+ cells (*p* < 0.0001; Figure 3d).

**Figure 4:**
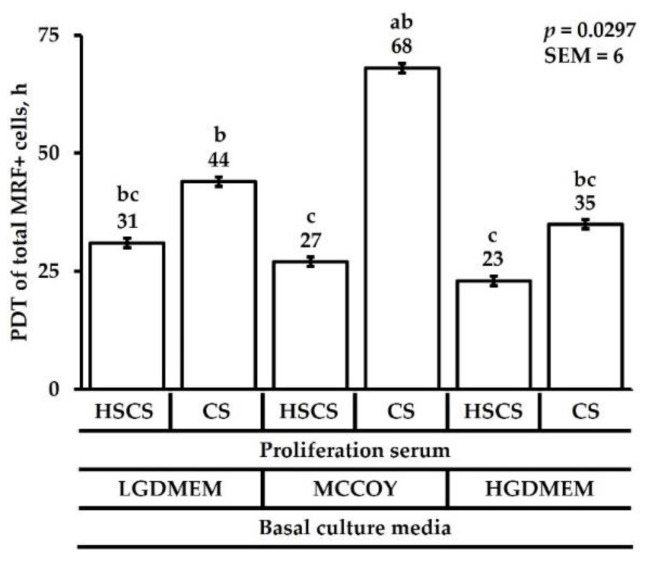
The effect of basal culture media and proliferation sera on population doubling time of total MRF+ cells expressed in h. Cells were cultured in either low glucose Dulbecco’s Modified Eagles medium (LGDMEM), McCoy’s 5A (MCCOY), or high glucose Dulbecco’s Modified Eagles medium (HGDMEM) supplemented with either 5% horse serum + 10% chicken serum (HSCS) or 15% chicken serum (CS). ^a–c^ Means with different superscripts describe a difference *p* < 0.05 and were generated from the pairwise mean comparison analysis.

The population doubling time of total MRF+ cells from 48 h post-plating until 96 h post-plating took less time to proliferate in both HGDMEM media, LGDMEM+HSCS media, and MCCOY+HSCS media (*p* = 0.0297; Figure 4). Even though the MCCOY+HSCS media had a lower doubling time, DMEM media produced more MRF+ cells than MCCOY media.

### 3.2. Differentiation Heterogeneity and Satellite Cell Fusion

After proliferation, primary SC heterogeneity of unfused SC and fused myonuclei were assessed after 96 h in differentiation media where cells were differentiated in their same respective media, but with one of two different differentiation sera. Culturing cells in HGDMEM media supplemented with HSCS at proliferation and CS at differentiation produced more fused myonuclei within myotubes without expressing any other MRF (*p* = 0.0002; Table 4), more MyoD+ nuclei within myotubes (*p* = 0.0108; Table 4), and more total myonuclei (*p* = 0.0007; Table 4) compared with other media and sera combinations noting that cells cultured in LGDMEM media with the same sera combination mentioned produced the second largest populations. However, the same media combinations also produced the most unfused MRF+ cells (*p* < 0.0001; Table 4) while basal culture media or sera at proliferation and differentiation did not impact total unfused MRF- or total unfused cells (*p* > 0.1081; Table 4). Cells cultured in MCCOY or media + HSCS at proliferation + CS at differentiation produced the most cells at the end of differentiation 96 h post-proliferation with LGDMEM with the same sera combination producing the second next highest population of total cells (*p* = 0.0410; Table 4).

**Figure 5:**
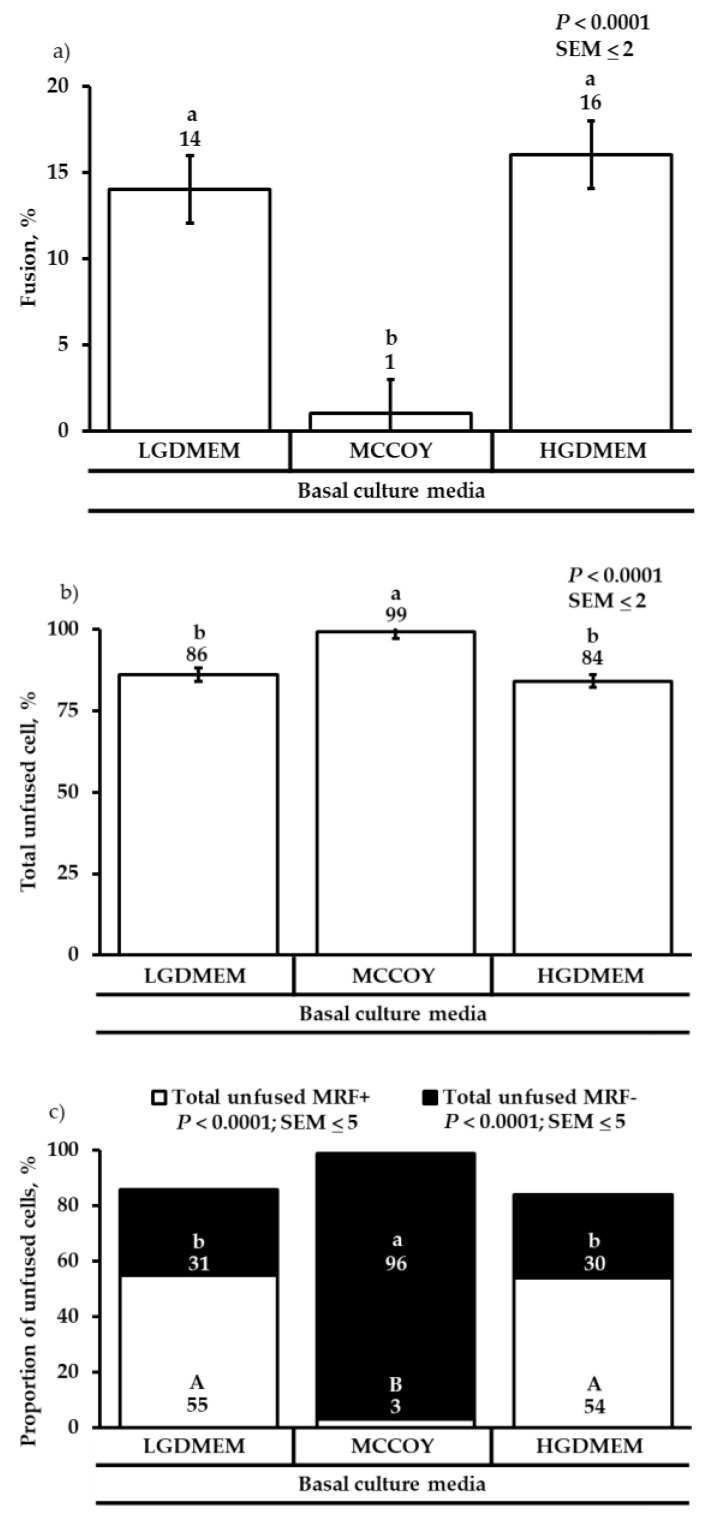
The effect of basal culture media fusion percentage (**a**), percentage of unfused satellite cells (**b**), and proportions of unfused MRF+/MRF- cells to total cells (**c**). Cells were cultured in either low glucose Dulbecco’s Modified Eagles medium (LGDMEM), McCoy’s 5A (MCCOY), or high glucose Dulbecco’s Modified Eagles medium (HGDMEM). ^a,b; A,B^ Means with different superscripts describe a difference *p* < 0.05 and were generated from the pairwise mean comparison analysis.

Even though MCCOY + HSCS + CS media produced a large number of cells, MCCOY media as a main effect failed to differentiate cells compared with DMEM cultured cells, which were revealed to have a higher fusion percentage of fused SC (*p* < 0.0001; Figure 5a). This data indicates that 99% of cells cultured with MCCOY media were unfused cells compared with 84–86% of cells being unfused when cultured in DMEM media (*p* < 0.0001; Figure 5b). Of the 99% that remain to be unfused in MCCOY media, 96% of the cells were MRF- and 3% were MRF+ unfused cells (*p* < 0.0001; Figure 5c). Therefore, cells cultured with either LGDMEM or HGDMEM media produced 84–86% of unfused cells where 54–55% were MRF+ cells and 30–1% were MRF- cells (*p* < 0.0001; Figure 5c) indicating the ability of DMEM media to support myogenic cells compared with supporting less DAPI+ only cells.

## 4. Discussion

Primary satellite cell cultures may provide a useful model to understand SC function as it relates to muscle growth in production animals, such as chickens. While C2C12 and L6 SC lines commonly exist for studying mammalian SC function, lines for broiler chickens are not common, and thus researchers utilize primary SC cultures when studying muscle growth in broiler chickens. However, studies to optimize SC culture conditions in terms of media and sera combinations have not been published unlike media conditions for turkey SC performed by McFarland and colleagues in 1988 [11].

There are inconsistencies in the literature regarding culture media for primary SC cultures; however, many researchers utilize a HGDMEM media for plating and then switch to a MCCOY media for proliferation containing HSCS before being switched to a 3% HS + DMEM culture media containing other components or just the HS [13,15]. In this experiment, a switch of media was not performed, and SC remained in their same basal culture media for the entirety of the experiment. Regarding basal culture media, the only difference between the composition of LGDMEM and HGDMEM is the D-glucose concentration where LGDMEM has a concentration of 1000 mg per L and HGDMEM has a concentration of 4500 mg per L of D-glucose. The MCCOY media has a D-glucose concentration of 3000 mg per L and additional components in the media, such as additional amino acids and vitamins (Supplemental Table S1). In this experiment, the difference in media composition and sera may explain the differences observed during proliferation where MCCOY+HSCS produced the most cells compared with the other media and sera combinations. However, the additional amino acids and vitamins may also explain why MRF- populations were supported more in MCCOY+HSCS media as DMEM media lacked the additional amino acids and vitamins. Furthermore, circulating chicken glucose was reported to be between 2000 and 5000 mg per L [18], which fits well within the parameters of the media utilized in this experiment. However, data suggest that high glucose concentrations may induce SC lipogenesis [19,20]. Recently, data suggest that excess glucose can even impede SC proliferation when comparing a serum-free DMEM and HGDMEM culture media [21]. While our data are not consistent with Furuichi and colleagues, as they do not reveal decreased proliferation in higher glucose containing basal media, they may indicate that the use of LGDMEM is more optimal than the HGDMEM media as both DMEM media supported MRF+ cells in proliferation and fusion of SC in differentiation. Our data are in agreement with Dodson and others’ work in ovine SC where MCCOY media better supported cell proliferation compared with other media, such as LGDMEM, while LGDMEM better supported SC fusion [9].

In McFarland’s work, regardless of any media utilized in the experiment, chicken serum produced more turkey SC during proliferation compared with other sera studied [11]. In this study, we did not compare just HS or CS in proliferation where a combination of HSCS was utilized and compared with just CS. It was revealed that the combination of HS and CS in proliferation supported total DAPI+ cell and total MRF+ cells by 96 h post-plating compared with 48 and 72 h post-plating. McFarland and others also revealed that a switch from MCCOY to HGDMEM from proliferation to differentiation increased SC fusion [11]. In this experiment, the other factor switched from proliferation to differentiation was the differentiation sera and not the basal culture media. This may indicate why we have a low fusion percentage of 14–16% in the DMEM cultured cells because a switch in media was not implemented. The work of McFarland may be the foundation of the current culture media seen in the current literature regarding primary cell culture in avian species; however, McFarland utilized turkey SC and not broiler chicken SC. Furthermore, the broiler chicken genetics have changed over time due to the improvements in genetics, feed formulations, and management resulting in current genetics having improved muscle growth compared to past genetics [22,23,24,25,26]. With regard to bovine SC culture media, researchers have also shown that different sera source can impact SC fusion [6]. Their data revealed how HS improved SC fusion compared with fetal calf serum and sheep serum. While our study did not assess calf or sheep serum, data suggest that the use of CS during differentiation may be optimal as it supports SC fusion compared with HS.

## 5. Conclusions

In conclusion, primary broiler chicken SC proliferated in MCCOY media produced the most cells. However, DMEM cultured cells produced more MRF+ cells when supplemented with HSCS. The proportion of MRF+ cells was also greater in DMEM cultured cells compared with MCCOY media. The glucose concentration of LGDMEM may better support SC proliferation based on the published literature by other researchers. When cells were switched to differentiation media, MCCOY media failed to differentiate cells compared with DMEM cultured cells. Furthermore, the supplementation of CS at differentiation produced more myonuclei compared with HS. Therefore, LGDMEM + HSCS during proliferation and CS during differentiation appear to be optimal for culturing primary broiler chicken SC to better understand their function in broiler chicken muscle growth.

## Figures and Tables

**Table 1:** The effect of basal culture media and proliferation serum on proliferating satellite cells heterogeneity at 48 h post-plating.

Cell Populations ^3^	Basal Culture Media ^1^						SEM ^4^	*p*-Value
	LGDMEM		MCCOY		HGDMEM			
	Proliferation Serum ^2^							
	HSCS	CS	HSCS	CS	HSCS	CS		
Myf-5+	1	1	1	1	1	0	1	0.8210
MyoD+	-	-	-	-	-	-	-	-
Pax7+	18	27	17	10	24	12	5	0.1442
Myf-5+:MyoD+	1	1	4	1	0	1	1	0.1162
Myf-5+:Pax7+	39	28	26	21	35	38	4	0.2069
MyoD+:Pax7+	0	0	1	0	1	0	0.3	0.1004
Myf-5+:MyoD+:Pax7+	23 ^yz^	19 ^yz^	45 ^x^	25 ^y^	23 ^yz^	16 ^z^	4	0.0911

^1^ Primary satellite cells were cultured in either low glucose Dulbecco’s Modified Eagles medium (LGDMEM), McCoy’s 5A (MCCOY) and high glucose Dulbecco’s Modified Eagles medium (HGDMEM) during proliferation. ^2^ Media was supplemented with either 5% horse serum + 10% chicken serum (HSCS) or 15% chicken serum (CS). ^3^ After 48 h post-plating, cells were indirect immunofluorescence stained for three myogenic regulatory factors (MRF), heterogeneity of all single, double, and triple positive MRF expressing cells were enumerated, and expressed on a mm^2^ basis. ^4^ SEM = highest standard error of the LS means. ^x–z^ Means with different superscripts describe a tendency 0.0501 ≤ *p* ≤ 0.10 and were generated from the pairwise mean comparison analysis.

**Table 2:** The effect of basal culture media and proliferation serum on proliferating satellite cell heterogeneity at 72 h post-plating.

Cell Populations ^3^	Basal Culture Media ^1^						SEM ^4^	*p*-Value
	LGDMEM		MCCOY		HGDMEM			
	Proliferation Serum ^2^							
	HSCS	CS	HSCS	CS	HSCS	CS		
Myf-5+	1	1	1	0	1	1	1	1.000
MyoD+	-	-	-	-	-	-	-	-
Pax7+	18 ^b^	22 ^b^	43 ^a^	11 ^b^	16 ^b^	18 ^b^	6	0.004
Myf-5+:MyoD+	26 ^a^	8 ^b^	8 ^b^	3 ^b^	27 ^a^	8 ^b^	3	0.015
Myf-5+:Pax7+	32	16	15	4	22	19	3	0.1191
MyoD+:Pax7+	1	1	2	0	1	0	1	0.3200
Myf-5+:MyoD+:Pax7+	126 ^b^	79 ^d^	206 ^a^	92 ^cd^	119 ^bc^	117 ^bc^	12	<0.0001

^1^ Primary satellite cells were cultured in either low glucose Dulbecco’s Modified Eagles medium (LGDMEM), McCoy’s 5A (MCCOY) and high glucose Dulbecco’s Modified Eagles medium (HGDMEM) during proliferation. ^2^ Media was supplemented with either 5% horse serum + 10% chicken serum (HSCS) or 15% chicken serum (CS). ^3^ After 72 h post-plating, cells were indirect immunofluorescence stained for three myogenic regulatory factors (MRF), heterogeneity of all single, double, and triple positive MRF expressing cells were enumerated, and expressed on a mm^2^ basis. ^4^ SEM = highest standard error of the LS means. ^a–d^ Means with different superscripts differ *p* ≤ 0.05 and were generated from the pairwise mean comparison analysis.

**Table 3:** The effect of basal culture media and proliferation serum on proliferating satellite cell heterogeneity at 96 h post-plating.

Cell Populations ^3^	Basal Culture Media ^1^						SEM ^4^	*p*-Value
	LGDMEM		MCCOY		HGDMEM			
	Proliferation Serum ^2^							
	HSCS	CS	HSCS	CS	HSCS	CS		
MYF5+	2	2	1	1	4	1	1	0.0986
MYOD+	-	-	-	-	-	-	-	-
PAX7+	28	16	66	21	75	23	11	0.1640
MYF5+:MYOD+	34 ^b^	9 ^c^	21 ^bc^	6 ^c^	78 ^a^	9 ^c^	6	0.0001
MYF5+:PAX7+	56	33	17	6	65	29	7	0.2297
MYOD+:PAX7+	2	7	1	2	0	1	3	0.6778
MYF5+:MYOD+:PAX7+	224	130	287	105	261	140	19	0.0572

^1^ Primary satellite cells were cultured in either low glucose Dulbecco’s Modified Eagles medium (LGDMEM), McCoy’s 5A (MCCOY) and high glucose Dulbecco’s Modified Eagles medium (HGDMEM) during proliferation. ^2^ Media was supplemented with either 5% horse serum + 10% chicken serum (HSCS) or 15% chicken serum (CS). ^3^ After 96 h post-plating, cells were indirect immunofluorescence stained for three myogenic regulatory factors (MRF), heterogeneity of all single, double, and triple positive MRF expressing cells were enumerated, and expressed on a mm^2^ basis. ^4^ SEM = highest standard error of the LS means. ^a–c^ Means with different superscripts differ *p* ≤ 0.05 and were generated from the pairwise mean comparison analysis.

**Table 4:** The effect of basal culture media, proliferation serum, and differentiation serum on differentiating satellite cell heterogeneity at 96 h post-proliferation.

Cell Populations ^4^	Basal Culture Media ^1^												SEM ^5^	*p*-Value
	LGDMDM				MCCOY				HGDMEM					
	Proliferation Serum ^2^													
	HSCS		CS		HSCS		CS		HSCS		CS			
	Differentiation Serum ^3^													
	HS	CS	HS	CS	HS	CS	HS	CS	HS	CS	HS	CS		
Fused myonuclei														
Myonuclei: MRF-	10 ^d^	81 ^b^	16 ^cd^	18 ^cd^	2 ^d^	1 ^d^	1 ^d^	1 ^d^	16 ^dc^	130 ^a^	8 ^cd^	35 ^c^	8	0.0002
MYOD+	0 ^b^	3 ^b^	0 ^b^	0 ^b^	0 ^b^	0 ^b^	0 ^b^	0 ^b^	0 ^b^	10 ^a^	3 ^b^	0 ^b^	2	0.0108
PAX7+	10	27	6	27	1	1	0	0	5	29	4	39	6	0.8192
MYOD+:PAX7+	0	1	1	0	0	0	0	0	0	6	0	1	1	0.2266
Total myonuclei	20 ^de^	112 ^b^	23 ^de^	45 ^d^	3 ^e^	2 ^e^	1 ^e^	1 ^e^	21 ^de^	175 ^a^	15 ^e^	75 ^c^	9	0.0007
Unfused cell populations														
MYOD+	-	-	-	-	-	-	-	-	-	-	-	-	-	-
PAX7+	78 ^bc^	380 ^a^	57 ^cd^	139 ^b^	27 ^cd^	49 ^cd^	9 ^d^	9 ^d^	79 ^bc^	441 ^a^	61 ^cd^	133 ^b^	22	<0.0001
MYOD+:PAX7+	-	-	-	-	-	-	-	-	-	-	-	-	-	-
Total unfused MRF+	78 ^cd^	380 ^b^	57 ^de^	139 ^c^	27 ^de^	10 ^de^	9 ^e^	9 ^e^	79 ^cd^	443 ^a^	61 ^de^	133 ^c^	22	<0.0001
Total unfused MRF-	29	436	13	396	684	965	374	478	20	508	12	257	71	0.5308
Total unfused cells	107	815	70	535	711	1015	383	487	99	952	73	389	61	0.1081
Total DAPI+ cells	127 ^f^	927 ^b^	93 ^f^	580 ^cd^	714 ^c^	1017 ^ab^	384 ^e^	488 ^de^	119 ^f^	1126 ^a^	87 ^f^	463 ^de^	62	0.041

^1^ Primary satellite cells were cultured in either low glucose Dulbecco’s Modified Eagles medium (LGDMEM), McCoy’s 5A (MCCOY) and high glucose Dulbecco’s Modified Eagles medium (HGDMEM) during proliferation. ^2^ Media was supplemented with either 5% horse serum + 10% chicken serum (HSCS) or 15% chicken serum (CS) during proliferation. ^3^ Media was supplemented with 3% horse serum (HS) or 3% chicken serum (CS) at differentiation. ^4^ After 96 h post-proliferation, cells were indirect immunofluorescence stained for two myogenic regulatory factors (MRF) and a nascent myotube marker. Heterogeneity of all single and double positive MRF expressing cells within myotubes or unfused cells were enumerated and expressed on a mm^2^ basis. ^5^ SEM = highest standard error of the LS means. ^a–f^ Means with different superscripts differ *p* ≤ 0.05 and were generated from the pairwise mean comparison analysis.

## Data Availability

All datasets generated for this study are included in the manuscript/Supplemental Material.

## Supplementary Materials

The following supporting information can be downloaded at: https://www.mdpi.com/article/10.3390/ani12111425/s1, Table S1 Basal culture media composition and Figure S1 Representative images of primary broiler chicken satellite cells cultured in different basal culture media for 96 h in proliferation media.
