# Supplementary material for: Effect of Different Basal Culture Media and Sera Type Combinations on Primary Broiler Chicken Muscle Satellite Cell Heterogeneity during Proliferation and Differentiation
Source: Animals (Basel). 2022 May 31;12(11):1425. doi: 10.3390/ani12111425 (PMC9179426; doi:10.3390/ani12111425)
Supplement: Supplementary file 1 [file animals-12-01425-s001.zip › animals-1715453-supplementary.pdf]

Supplemental Table S1. Basal culture media composition

Components[2]	Basal culture media[1]		
	LGDMEM	MCCOY	HGDMEM
Amino Acids			
Glycine	30	7.5	30
L-Alanyl-glutamine	862	-	862
L-Arginine hydrochloride	84	42.1	84
L-Cystine	48	-	63
L-Histidine hydrochloride-H2O	42	20.96	42
L-Isoleucine	105	39.36	105
L-Leucine	105	39.36	105
L-Lysine hydrochloride	146	36.5	146
L-Methionine	30	14.9	30
L-Phenylalanine	66	16.5	66
L-Serine	42	26.3	42
L-Threonine	95	17.9	95
L-Tryptophan	16	3.1	16
L-Tyrosine	72	18.1	104
L-Valine	94	17.6	94
Vitamins			
Choline chloride	4	5	4
D-Calcium pantothenate	4	0.2	4
Folic acid	4	10	4
Niacinamide	4	0.5	4
Pyridoxine hydrochloride	4	0.5	4
Riboflavin	0.4	0.2	0.4
Thiamine hydrochloride	4	0.2	4
i-Inositol	7.2	36	7.2
Inorganic Salts			
Calcium chloride (CaCl2-2H2O)	264	100	200
Ferric nitrate (Fe(NO3)3"9H2O)	0.1	-	0.1
Magnesium sulfate (MgSO4-7H2O)	200	200	97.67
Potassium chloride (KCl)	400	400	400
Sodium bicarbonate (NaHCO3)	3700	2200	3700
Sodium chloride (NaCl)	6400	6460	6400
Sodium phosphate monobasic (NaH2PO4-2H2O)	141	580	125
Other Components			
D-Glucose (Dextrose)	1000	3000	4500
Phenol red	15	10	15
Sodium pyruvate	110	-	110

Components[2]	Basal culture media[1]		
	LGDMEM	MCCOY	HGDMEM
Additional Amino Acids			
L-Alanine	-	13.9	-
L-Asparagine	-	45	-
L-Aspartic acid	-	19.97	-
L-Cysteine	-	31.5	-
L-Glutamic Acid	-	22.1	-
L-Glutamine	-	219.2	-
L-Hydroxyproline	-	19.7	-
L-Proline	-	17.3	-
Additional Vitamins	-		-
Ascorbic acid	-	0.5	-
Biotin	-	0.2	-
Nicotinic acid (Niacin)	-	0.5	-
Para-aminobenzoic acid	-	1	-
Pyridoxal hydrochloride	-	0.5	-
Vitamin B12	-	2	-
Additional Other Compounds	-		-
Bacto-peptone	-	600	-
Glutathione (reduced)	-	0.5	-

[1]Basal culture media include low-glucose Dulbecco's Modified Eagle's Medium (LGDMEM), McCoy's 5A (MCCOY), and high-glucose Dulbecco's Modified Eagle's Medium. [2]Media components are expressed in mg/L.

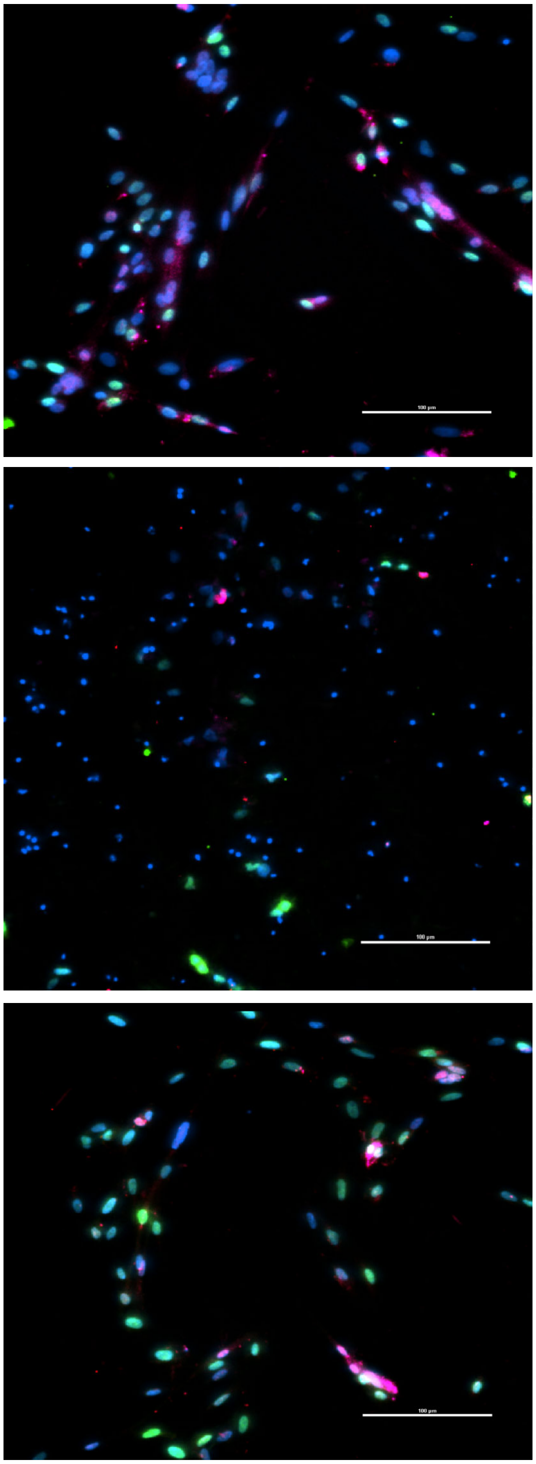

A LGDMEM

B McCoy's

C HGDMEM

Supplemental Figure S1. Representative images of myogenic regulatory factor expression in broiler chicken satellite cells (SC) after 96 h post-plating in 1 of 3 different basal media ((A) Low glucose Dulbecco's Modified Eagle's (LGDMEM); (B) McCoy's 5A; (C) High glucose Dulbecco's Modified Eagle's media (HGDMEM)) supplemented with 5% horse serum + 10% chicken serum. Panels A-C: nuclei (blue, DAPI+), Pax7+ SC (green), MyoD+ SC (red), and Myf5+ SC (purple). Scale bar = 100 μm.
